# Metabolic Profiling during Acute Myeloid Leukemia Progression Using Paired Clinical Bone Marrow Serum Samples

**DOI:** 10.3390/metabo11090586

**Published:** 2021-08-31

**Authors:** Hyun Kyu Kim, Su Young Son, Jae Sang Oh, Ye Na Song, Ja Min Byun, Youngil Koh, Junshik Hong, Sung-Soo Yoon, Choong Hwan Lee, Dong-Yeop Shin, Man Ryul Lee

**Affiliations:** 1 Soonchunhyang Institute of Medi-Bio Science (SIMS), Soon Chun Hyang University, Cheonan 31151, Korea; hyunkyu8505@naver.com (H.K.K.); thddpsk123@naver.com (Y.N.S.); 2 Department of Bioscience and Biotechnology, Konkuk University, Seoul 05029, Korea; syson119@naver.com; 3 Department of Neurosurgery, College of Medicine, Cheonan Hospital, Soonchunhyang University, Cheonan 31151, Korea; metatron1324@naver.com; 4 Department of Internal Medicine, Division of Hematology and Medical Oncology, Seoul National University Hospital, Seoul 03080, Korea; jaminbyun@naver.com (J.M.B.); go01@snu.ac.kr (Y.K.); alertjun@hanmail.net (J.H.); ssysmc@snu.ac.kr (S.-S.Y.); 5 Center for Medical Innovation, Biomedical Research Institute, Seoul National University Hospital, Seoul 03080, Korea; 6 Cancer Research Institute, Seoul National University Hospital, Seoul 03080, Korea; 7 Research Institute for Bioactive-Metabolome Network, Konkuk University, Seoul 05029, Korea

**Keywords:** acute myeloid leukemia, cell metabolism, bone marrow serum, metabolomics

## Abstract

Cellular metabolic changes reflect the characteristics of patients with acute myeloid leukemia (AML) caused by genetic variations, which are important in establishing AML treatment. However, little is known about the metabolic profile of patients with genetic variation-induced AML. Furthermore, the metabolites differ with disease progression. Here, metabolites in the bone marrow serum of ten patients with AML and healthy individuals were analyzed using gas chromatography–mass spectrometry. Compared with that in healthy individuals, expression of most metabolites decreased in patients with AML; hydroxylamine, 2-hydroxybutyric acid, monomethylphosphate, and ethylphosphate expression was unusually increased in the patients. We further examined serial metabolite changes across the initial diagnosis, postremission, and relapse phases. Patients with relapse showed increased metabolite expression compared with those in the diagnostic phase, confirming that patients with AML had aggressively modified leukemic cells. However, a clear difference in metabolite distribution was not observed between the diagnosis and complete remission phases, suggesting that the metabolic microenvironment did not change significantly despite complete remission. Interestingly, metabolite profiles differed with genetic variations in leukemic cells. Our results, which were obtained using paired samples collected during AML progression, provide valuable insights for identifying vulnerable targets in the AML metabolome and developing new treatment strategies.

## 1. Introduction

Acute myeloid leukemia (AML) is a malignant hematologic disease with a high risk of fatality. Although a highly intensive treatment strategy involving the combination of induction, consolidation, and allogeneic hematopoietic stem cell transplantation has been applied in patients with AML for more than 30 years, the prognosis of AML remains poor.

Massive genomic research on cancerous samples has provided us with the important information that every cancer has oncogenic driver mutations [1,2], which has resulted in the era of precision medicine. Chronic myeloid leukemia, another form of leukemia that is extremely homogeneous in view of pathogenic genetic aberrations, is representative of the dramatic success with molecular targeted agents [3], whereas no such advancements have been observed for AML partially due to its high heterogeneity. Fms-like tyrosine kinase 3 (FLT3) inhibitors, which target the most commonly mutated genes in AML, and Bcl-2 inhibitors, which target a universal oncogenic signaling pathway, are the first molecular targeted drugs developed in precision medicine with respect to AML treatment [4,5], along with many other drugs. These drugs have now changed the clinical landscape of AML treatment, but determining the cure for AML still has a long way to go.

Similar to other cancers, AML shows obvious genetic mutations and reprogramed nutrient acquisition and metabolic pathways to meet the needs of bioenergy, biosynthesis, and oxidation reduction [6]. In general, cancer cells metabolize ATP by converting pyruvate to lactate, rather than through the tricarboxylic acid cycle or mitochondrial oxidation [7,8], to meet the increased nutritional demand. Recently, however, it has been found that aerobic metabolism of sugars and enzymes is actively involved in cancer cell growth and division [9]. Each cell contains different types of metabolites because of the presence of individualized metabolic mechanism based on the fate of the cell. Thus, metabolic profiling has attracted considerable attention as an approach that can immediately detect dynamic cell changes and conditions, as well as early development [10]. Oncogenic mutations recurrently found in AML drive metabolic dysregulation, and dysregulated metabolism is closely linked to oncogenic addiction and thus potential therapeutic targets for AML. For example, mutation in the most common oncogene in AML, i.e., *FLT3*, is known to be associated with glycolysis [10]. Other oncogenic driver mutations, such as those in *MYC* and *RAS*, have been studied to drive metabolic reprogramming, including increased glycolysis, glutaminolysis, lipid synthesis, and mitochondrial biogenesis, which are important for AML cell proliferation and survival [11,12,13]. Furthermore, AML cells can hinder glucose uptake by normal tissues through desensitization to insulin by increasing the levels of serum insulin-like growth factor binding protein 1 [14]. Oxidative phosphorylation is activated in AML cells as compared to normal hematopoietic stem/progenitor cells [10]. Thus, AML cells have metabolic vulnerabilities depending on specific bioenergy sources [15]. The bone marrow microenvironment might experience dynamic changes in metabolic components based on the leukemic progression and chemotherapeutic agents during active induction chemotherapy. Accordingly, the vulnerability of leukemic cells, which can be a target of anticancer therapy, may also change continuously. However, such serial changes in patients with AML have not yet been investigated.

Based on metabolomics analyses of AML cells, we reasoned that metabolic profiling of bone marrow (BM) serum samples obtained from patients with AML could lead to a detailed understanding of the altered metabolism in the leukemic microenvironment. Skewed metabolism, including glutaminolysis, glycolysis, and lipid biosynthesis, could lead to the depletion of specific metabolites in the AML microenvironment. Recently, the concept of targeting the tumor microenvironment has become an inevitable aspect of decoding cancer cell survival [16].

Herein, we performed a metabolomics analysis in BM-derived serum samples of patients with AML at the time of initial diagnosis, postremission therapy, and relapse to investigate the changes in AML metabolome based on the treatment course and disease progression. Our findings could shed light on identifying potential therapeutic targets in the AML metabolome and devising appropriate treatment strategies.

## 2. Results

### 2.1. Patients’ Characteristics

In total, 10 patients diagnosed with AML were enrolled in this study. The median age of patients with AML was 54 years (range, 18–68 years), and half of the patients were female. RUNX1-RUNX1T1 translocation was detected in three patients, and internal tandem duplication (ITD) or tyrosine kinase domain (TKD) mutations in *FLT3* (hereafter referred to as FLT-ITD and FLT-TKD, respectively) were detected in four patients. The percentage of leukemic blasts among the total nucleated cells was 68.4% (range 20.4–84.7%) (Tables S1 and S3). Most patients (80%) were treated with standard induction chemotherapy (seven days of cytarabine arabinoside and three days of idarubicin), and 70% of the patients achieved complete remission (CR) after the first cycle of induction treatment. All patients in this study experienced relapse, with a median time to relapse of 11.3 months. Salvage chemotherapies of various combinations were applied to patients with AML relapse (Table S2).

### 2.2. Metabolic Differences in BM Microenvironment between Patients with AML and Healthy Individuals

**Figure 1:**
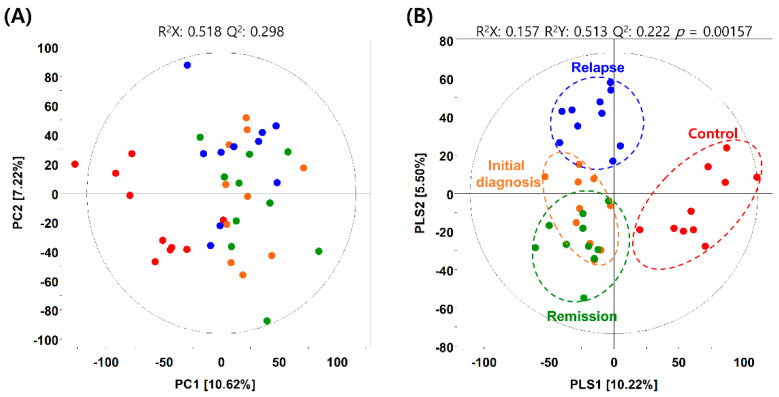
Metabolic differences between patients with acute myeloid leukemia (AML) and healthy individuals. (**A**) Principal component analysis (PCA) and (**B**) partial least squares–discriminant analysis (PLS-DA) score plots obtained using the dataset from gas chromatography–time of flight–mass spectrometry analysis of serum samples from healthy individuals (control) and patients with AML. Red dots, control group; orange dots, initial diagnosis group; green dots, remission group; blue dots, relapse group.

The metabolites in the BM-derived serum samples of patients with AML were analyzed using gas chromatography–time of flight–mass spectrometry (GC–TOF–MS), and 19,210 mass spectral variables were used for further multivariate analysis of each feature. The relative number of metabolites in each patient group was quantified and compared with that in all groups combined. We performed principal component analysis (PCA) and partial least squares–discriminant analysis (PLS–DA) to visualize the metabolic differences between patients with AML and healthy individuals. The PCA identified the distribution of metabolites between healthy individuals and patients with AML but did not show a marked difference between patients in the initial diagnosis and those in the CR or relapse phases (Figure 1A). To analyze variables that were not reflected in the PCA, PLS–DA was conducted to determine the distribution of metabolites within each sample. The PLS–DA, with a model value of R^2^X (cum) = 0.214, R^2^Y (cum) = 0.587, and Q^2^ (cum) = 0.185, displayed the model’s suitability and prediction accuracy. PLS–DA showed that the metabolite distribution in patients with AML relapse was clearly distinct from that in patients initially diagnosed with AML or in remission (Figure 1B and Figure S1). Unexpectedly, the metabolite distribution in patients in the initial diagnosis and remission phases showed unclear distinction, which suggested that the BM microenvironment in patients with AML did not change significantly even after achieving CR.

Based on the PLS–DA model, variable importance in projection (VIP) scores, which differed with the patient group, greater than 1.0 were used for metabolic analyses. VIP scores are important parameters for detecting the probable pathways, including potential metabolic marker candidates and diseases, which reflect the correlation between various biological states and metabolites. Statistical significance was determined using *p* < 0.05, which was derived from a Student’s *t*-test [17]. The selected metabolites were identified by comparing the mass spectral fragment patterns with the commercial standard compounds, and the National Institutes of Standards and Technology library, Human Metabolome Database (http://www.hmdb.ca/; accessed on 12 June 2021), and a variety of other databases. A total of 40 metabolites, including 17 amino acids, 4 organic acids, 6 fatty acids/lipids, 2 sugars/sugar alcohols, and 5 unknown metabolites, were identified with VIP > 1.0 as metabolites that differed significantly among the experimental groups (Table 1).

### 2.3. Hierarchical Clustering of Metabolites among the AML Initial Diagnosis, Relapse, and Remission Groups

**Figure 2:**
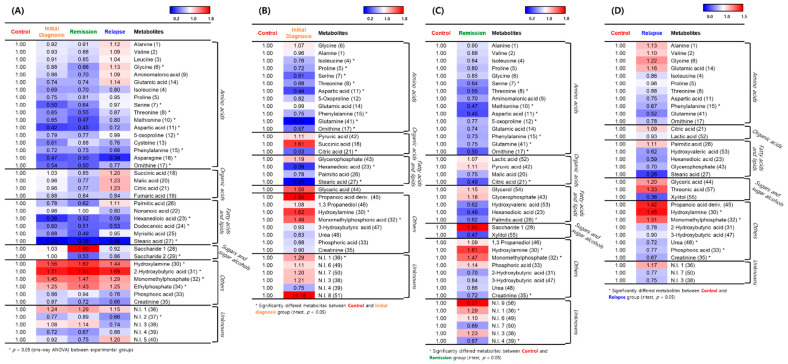
Representative heatmaps for the relative contents of significantly different metabolites between the healthy individuals and patients with acute myeloid leukemia (AML). (**A**) Differences between the control group and the initial diagnosis, remission, and relapse patient groups. (**B**) Differences between the control and initial diagnosis groups. (**C**) Differences between the control and remission groups. (**D**) Differences between the control and relapse groups. The colored squares (blue-to-red) represent fold changes normalized by the average content of each metabolite in the control group. The color scheme is as follows: lower limit value, blue; middle value (1.0), white; and upper limit value, red. * means that metabolites significantly differed between the control and patient group.

Expression of many metabolites was decreased in patients with AML compared with healthy individuals, whereas levels of hydroxylamine, 2-hydroxybutyric acid, monomethylphosphate, and ethylphosphate were elevated in patients with AML (Figure 2A). To determine the expression of metabolites based on the degree of leukemia progression, we performed an orthogonal partial least squares discriminant analysis (OPLS–DA) of the control and experimental groups. The OPLS–DA score plot derived from GC–TOF–MS dataset showed distinct patterns by OPLS1 (18.68%) (Figure S2a). Based on this model, the metabolites that differed significantly between the control and initial diagnosis group were selected using the variable importance in projection value (VIP > 1.0). A heatmap was generated to analyze the patterns of changes in the relative metabolite content for each metabolite family, showing a difference in expression between the groups. The relative metabolite content in the experimental groups was presented in terms of fold change with respect to that in the control group (Figure 2B). Levels of amino acids, except for glycine, alanine, and glutamic acid, tended to decrease in the initial diagnosis group compared with the control group. The degree of decrease was the highest for glutamine. Levels of fatty acids and lipids, except for glycerophosphate, tended to be decreased in all patient groups compared with the control group, with the level of stearic acid being the most reduced. Levels of pyruvic acid and succinic acid, as organic acids, tended to increase, whereas that of citric acid tended to decrease in all patient groups compared with the control group. Propanoic acid, hydroxylamine, and monomethylphosphoric acid levels tended to increase in the initial diagnosis group compared with the control group.

According to the OPLS–DA of the control and remission groups, there was a significant difference of 16.46% in the expression of metabolites (Figure S2b). Based on this model, different metabolites were screened (VIP > 1.0). Levels of all amino acids were reduced in the serum of patients in the remission group compared with that in healthy individuals in the control group. Levels of lactic acid and pyruvic acid (organic acids) and glycerol and glycerophosphate (fatty acids and lipids) were slightly increased in the serum of patients in the remission group compared with the individuals in the control group. Levels of saccharides (sugars and sugar alcohols), hydroxylamine, monomethylphosphate, and phosphoric acid were increased in the remission group compared with the control group (Figure 2C).

OPLS–DA of the control and relapse groups revealed a significant difference of 19.16% in metabolite expression (Figure S2c). Accordingly, different metabolites were screened (VIP > 1.0). The metabolite expression pattern in the relapse group was different from that in the remission and initial diagnosis groups, compared with the expression in the control group (Figure 2D). Concerning the initial diagnosis and remission groups, levels of amino acids were mostly reduced in these groups compared with the control group. Levels of amino acids in the relapse group were similar to those in the control group, whereas levels of some metabolites (alanine, valine, glycine, and glutamic acid) were increased in the relapse group compared with the control group. Levels of palmitic acid (fatty acids and lipids), glyceric acid and threonic acid (sugars and sugar alcohols), propanoic acid, hydroxylamine, and monomethylphosphate were increased in the serum of patients in the relapse group compared with that in the individuals in the control group. These results indicated that different expression characteristics of metabolites reflected the degree of AML progression and metabolic system change.

### 2.4. Serial Metabolic Changes from AML Diagnosis to Relapse

**Figure 3:**
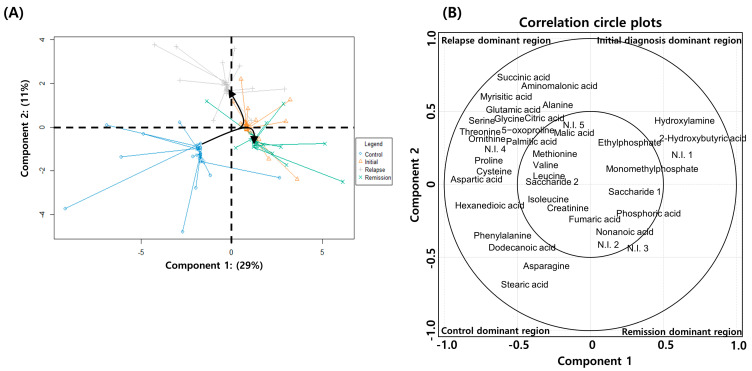
Spared partial least squares–discriminant analysis (sPLS–DA) of metabolite expression in patients with acute myeloid leukemia (AML) and healthy individuals. (**A**) sPLS–DA plot showing the combined distribution of metabolite expression in the initial diagnosis, remission, relapse, and healthy control groups. (**B**) Correlation circle plot displaying the correlation between the metabolite levels and disease progression. The metabolites in the inner circle of point are strongly correlated with the disease progression. The individual contribution of each metabolite to the progression of disease is represented at a distance away from the center in the correlation circle plots.

Non-paired analysis might lead to the loss of clinically relevant information originating from individual diversity. Therefore, we performed a paired-sample analysis, wherein each patient sample from the initial diagnosis, remission, and relapse groups in the study had serial paired samples for age/sex-matched healthy individuals. After performing PLS–DA on patients with AML, we additionally conducted spared PLS–DA (sPLS–DA) to determine if the metabolite profiles in patients significantly changed depending on their pathologic status. sPLS–DA enables the selection of the most predictive or discriminative features in the data to help classify the samples [18]. The analytical method helps predict a patient’s disease state using a selected variable. In addition, the calculation efficiency of sPLS–DA combined with graphic display confers stronger advantages in the multiclass case than the variable selection approach. Therefore, we applied sPLS–DA to determine whether the differential metabolites found in PLS–DA would be involved in changing the patients’ pathologic state. Samples from healthy individuals were distinguished from those of patients in the initial diagnosis, relapse, and remission groups by differential expression of 29% of the total metabolites (Component 1). Furthermore, patients in the initial diagnosis, relapse, and remission groups were predicted by differential expression of 11% of the total metabolites (Component 2) (Figure 3A). In the case of Component 1, the metabolite profile of the serum of patients in the remission group differed from that of individuals in the control group. In the case of Component 2, 11% of the total metabolites showed upregulated expression in the serum of patients in the initial diagnosis and relapse groups, whereas the remission and control groups showed similar characteristics. Therefore, it was found that the metabolites expressed after the initial diagnosis phase (component 2) were related to AML progression. Additionally, the metabolites contributing to each pathological state were identified after categorizing them into four quarters based on the zone representing each pathological state. The metabolites divided into four regions separated by the zero intersection point of each axis indicated a strong correlation between disease progression and metabolite levels (Figure 3A).

According to the analysis, healthy individuals are identifiable with the quantitative increases in the levels of hexanedioic acid, isoleucine, creatinine, phenylalanine, dodecanoic acid, asparagine, and stearic acid; patients in the initial diagnosis phase are identifiable by 2-hydroxybutyric acid, hydroxylamine, monomethylphosphate, and ethylphosphate; patients in remission are identifiable by nonanoic acid, saccharide 1, and phosphoric acid; and patients with relapse are identifiable by alanine, valine, leucine, proline, glycine, serine, threonine, aspartic acid, 5-oxoproline, cysteine, glutamic acid, ornithine, succinic acid, aminomalonic acid, malic acid, citric acid, myrisitic acid, palmitic acid, and saccharide 2. Metabolites contributing to each pathologic state showed significant and specific differences compared with those in the healthy individuals (Figure 3B). Therefore, these findings suggested that the BM-derived serum metabolite analysis could potentially predict AML diagnosis and prognosis.

### 2.5. Differences in Metabolite Profiles with Genetic Variations in AML

**Figure 4:**
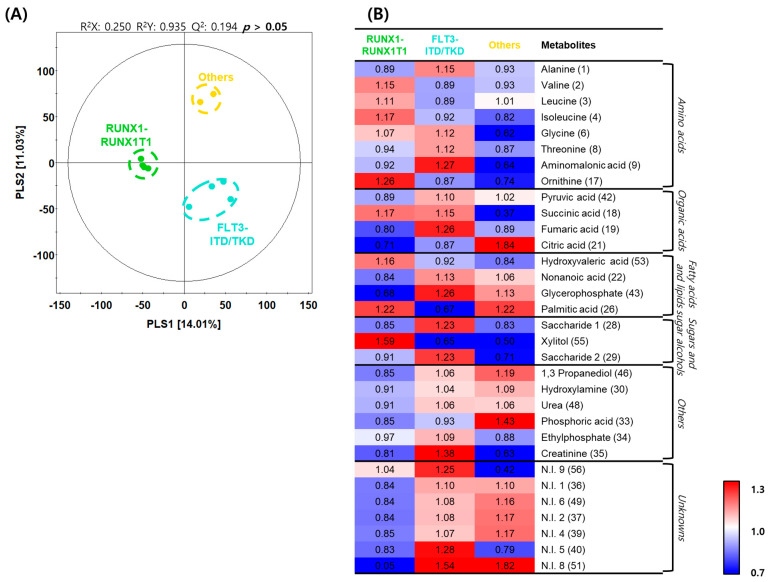
Differences in metabolite profiles based on genetic aberrations in acute myeloid leukemia. (**A**) Partial least squares–discriminant analysis (PLS–DA) score plots constructed using data from gas chromatography–time of flight–mass spectrometry analysis of the initial diagnosis patient subgroups. Green dots, RUNX1-RUNX1T1 group; light blue dots, Fms-like tyrosine kinase 3 (FLT3)-internal tandem duplication (ITD)/tyrosine kinase domain (TKD) group; and yellow dots, others group. (**B**) Heatmap analysis representing differential metabolites among the initial diagnosis subgroups with relative metabolite abundance. The relative level in the heatmap represents fold changes normalized to the average level of each metabolite. The color scheme is as follows: lower limit value (0.7), blue; middle value (1.0), white; and upper limit value (1.3), red.

We further classified patients in the initial diagnosis group into three experimental groups: the patient group with AML caused by RUNX1-RUNX1T1 translocation (n = 4), the group with AML caused by FLT3-ITD/TKD mutation (n = 4), and the group with AML induced by other causes. Through PLS–DA, the RUNX1-RUNX1T1 group and the other two groups were categorized according to PLS1 (14.01%), and the FLT3-ITD/TKD and the other two groups were categorized according to PLS2 (11.03%) (Figure 4A). Based on the PLS–DA model, metabolites in each group were analyzed, and eight amino acids, four organic acids, four fatty acids and lipids, and three sugars and sugar alcohols were detected as differentially expressed metabolites (Table 2). A heatmap was generated to analyze the patterns of changes in the relative metabolite content in each metabolite family. The changes in content were presented as the fold change with respect to the mean value of each metabolite. In the case of amino acids, levels of valine, leucine, isoleucine, and ornithine tended to be relatively higher in the RUNX1-RUNX1T1 group than in the other two groups; levels of alanine, glycine, threonine, and aminomalonic acid tended to be relatively higher in the FLT3-ITD/TKD group than in the other two groups. In the case of organic acids, the level of citric acid tended to be relatively higher in the FLT3-ITD/TKD group than in the other two groups; levels of pyruvic acid, succinic acid, and fumaric acid tended to be relatively higher in the FLT3-ITD/TKD group than in the other two groups; level of succinic acid tended to be relatively higher in the RUNX1-RUNX1T1 group than in the other two groups. In the case of fatty acids and lipids, the level of hydroxyvaleric acid tended to be relatively higher in the RUNX1-RUNX1T1 group than in the other two groups; levels of nonanoic acid and glycerophosphate tended to be relatively lower in the RUNX1-RUNX1T1 group than in the other two groups; the level of palmitic acid tended to be relatively lower in the FLT3-ITD/TKD group than in the other two groups. In the case of sugars and sugar alcohols, levels of saccharides 1 and 2 tended to be relatively higher in the FLT3-ITD/TKD group than in the other two groups; the level of xylitol tended to be relatively higher in the RUNX1-RUNX1T1 group than in the other two groups (Figure 4B).

## 3. Discussion

We selected 10 patients with AML and analyzed the changes in metabolites with respect to the AML progression state by using BM-derived serum samples obtained at initial diagnosis, during remission after chemotherapy, and after relapse. Our study revealed profound depletion in the levels of various amino acids and a few fatty acids in the BM microenvironment of patients newly diagnosed with AML. A similar phenomenon was observed at the time of CR after the initial induction chemotherapy. CR is an important clinical endpoint after induction chemotherapy, which predicts a good prognosis [19]. However, our study demonstrated that the metabolic and nutritional microenvironments of AML did not significantly change despite the achievement of CR. This highlights the easily forgotten fact that the state of CR is simply a log-scale reduction in the number of leukemic cells rather than an eradication of leukemic cells. Interestingly, a reversal of several amino acid depletions in the BM microenvironment of patients with AML relapse suggested that energy metabolism might change with clonal evolution at the time of relapse. Furthermore, glutamine and stearic acid were thought to be critical fuels for AML cells and continuously required by them, considering the fact that the levels of these two metabolites consistently decreased over the three states of disease progression. Hence, we postulate that therapeutic strategies targeting altered metabolism in newly diagnosed patients with AML and those with AML relapse should be discriminated based on the findings presented in this study.

A recent report suggested that metabolites have prognostic implications in AML, such that oncometabolite 2-hydroxyglutarate is associated with good prognosis, while phosphocholine and phosphoethanolamine are associated with poor prognosis. Overexpression of glutathione and alanine has been observed in chemoresistant patients [20]. Our study too showed different metabolic profiles between patients with AML harboring a favorable molecular marker, RUNX1-RUNX1T1, and those with a poor prognostic marker, FLT3-ITD/TKD. Levels of ornithine, palmitic acid, and xylitol were increased in the RUNX1-RUNX1T1 variation-induced AML patient group compared with the other patient groups. In the FLT3-ITD/TKD variation-induced AML patient group, levels of aminomalonic acid, fumaric acid, glycerophosphate, and saccharide were increased. Given that metabolites were expressed differently depending on the causative genetic alterations in AML, it is expected that the metabolite expression tendency would contribute to establishing a strategy for identifying the genetic causes and treatment of AML.

Previous studies have proposed that AML cells are dependent on glutamine for their survival and proliferation [21], which is consistent with our observation that glutamine was expressed irrespective of the disease status, i.e., treatment-naïve, post-remission, and relapse. Our results suggested that stearic acid was also depleted in the BM microenvironment of patients with AML, which is consistent with a previous study [20]. These findings support the notion that AML cells depend on glutaminolysis and fatty acid oxidation. Moreover, metabolic addiction of AML cells to specific metabolic pathways increases the probability of identifying novel therapeutic targets. The AML microenvironment commonly undergoes pan-depletion of metabolites that are used as an energy source, including glutamine and stearic acid, and further is deranged according to the genetic mutational profile and disease status of AML.

Traditionally, metabolites are generated during the final disease stage to maintain vital phenomena. By monitoring changes in the biological system in vivo, it is possible to provide dynamic snapshots for all physiological conditions, which cannot be analyzed through genetic expression and proteomics analyses alone. However, in a variety of metabolomics analyses of AML, it has been difficult to obtain consistent findings with respect to biomarkers, and their robustness has been dubious. Hence, in this study, by conducting a dynamic metabolite analysis of patients with AML in the initial diagnosis, postremission, and relapse stages, we offered more unswerving findings on the physiological conditions and biomarkers in patients with AML. In additional research, it will be necessary to compare the metabolite profiles in patients that fully recover through remission and those who relapse and succumb to the disease after remission. The patients who participated in this study died after initial diagnosis, remission, or relapse. The cell metabolism after remission failed to return to the normal state, and hence, the patients relapsed and succumbed to the disease. Moreover, the metabolite profile of the patients in relapse was different from that of healthy individuals or newly diagnosed patients. This indicates that the AML cells became increasingly malignant under relapse. In the future, a larger prospective study to elucidate appropriate target metabolites for treating AML should be undertaken.

In conclusion, our study suggests that developing a therapeutic strategy to target metabolic vulnerabilities in AML can be promising for potentiating leukemic cell death by removing energy sources from the BM niche.

## 4. Materials and Methods

### 4.1. Patient Sample Collection

In total, 10 patients initially diagnosed with AML and treated with chemotherapy, allogeneic hematopoietic stem cell transplantation, or both at Seoul National University Hospital were enrolled in this study. Patients with mixed phenotypes of acute leukemia and acute promyelocytic leukemia were excluded. Additionally, patients who did not receive active treatment, including chemotherapy, did not experience relapse, or were lost to follow-up were excluded. Serial BM-derived serum samples, collected at three time points from each patient (initial diagnosis: patient’s serum samples were first diagnosed with leukemia, remission: patient’s serum samples were after leukemia treatment, and relapse: patient’s serum sample when diagnosed with leukemia again), were identical in clinically paired diagnosis and were selected as well as prepared for metabolomics analysis. Another 10 BM-derived serum samples collected as staging work-ups of sex- and age (±2)-matched patients with stage I–II lymphomas, which were confirmed to not involve bone marrow lymphoma, were used as normal controls. We carefully selected control cases through a thorough review of clinical information and bone marrow reports. Only cases obtained prior to chemotherapy—to exclude the possibility of a change in bone marrow microenvironment due to chemotherapy as well as cases reporting normocellular marrow without involvement of hematologic diseases—were used as controls. BM-derived serum samples from patients with AML were collected and stored at −20 °C.

### 4.2. Sample Preparation for Metabolite Analysis

The extraction of serum metabolites was performed using the methods described by Park et al. [22]. Briefly, each serum sample (100 μL) was extracted with 100% methanol (1 mL) and 10 μL of internal standard solution (2-chlorophenylalanine, 1 mg/mL in water) using an MM400 mixer mill (Retsch^®^, Haan, Germany) at a frequency of 30 Hz for 10 min, followed by sonication for 10 min. Then, the extracted samples were incubated for 1 h at 4 °C and centrifuged at 13,000 rpm for 10 min at 4 °C. The supernatants were filtered using 0.2 μm polytetrafluorethylene filters (Chromdisc, Daegu, Korea). The filtered samples were completely dried using a speed vacuum concentrator (Biotron, Seoul, Korea). Dried samples were re-dissolved in 100% methanol to a final concentration of 1000 ppm (10 mg/mL).

### 4.3. GC–TOF–MS Analysis

GC–TOF–MS analysis was performed using an Agilent 7890A GC system (Agilent Technologies, Palo Alto, CA, USA) equipped with an L-PAL3 autosampler and Pegasus^®^ HT TOF-MS system (LECO Corp., St. Joseph, MI, USA). Metabolites were separated using an RTX-5MS column (30 m length × 0.25 mm inner diameter × 0.25 μm particle size, Restek Corp., St. Joseph, MI, USA) with a constant flow of helium (1.5 mL) as the carrier gas. For analysis, all dried samples were oximated with 50 μL of methoxyamine hydrochloride (20 mg/mL in pyridine) for 90 min at 30 °C and silylated with 50 μL of *N*-methyl-*N*-(trimethylsilyl) trifluoroacetamide for 30 min at 37 °C. The derivatized samples (1 μL) were injected into the GC column in splitless mode. The analytical program for sample analysis was adopted from our previous study [22]. Moreover, metabolite analysis was performed in a random manner to reduce bias and systematic errors.

### 4.4. Statistical Analysis

Mass spectral data processing and multivariate analysis were conducted as described in our previous study [22]. Raw data derived from GC–TOF–MS analysis were transformed into .cdf format using LECO Chroma TOF software (version 4.44, LECO Corp., St. Joseph, MI, USA), and data processing including digitalization, peak selection, alignment, peak intensity normalization, and baseline correction was conducted using the Metalign software (RIKILT-Institute of Food Safety, Wageningen, The Netherlands). The processed data were exported into Excel files (Microsoft Corp., Redmond, WA, USA). PCA, PLS–DA, and OPLS–DA were performed to compare the metabolite profiles between the control and AML patient groups using SIMCA-P+ software (version. 12.0; Umetrics, Umea, Sweden). The significance of PLS–DA and OPLS–DA models was determined by analysis of variance testing of cross-validated predictive residuals. Discriminative metabolites were selected based on the VIP scores of the PLS–DA and OPLS–DA models. The different metabolites obtained from GC–TOF–MS analysis were searched against various databases, including the National Institute of Standards and Technology database (Version 2.0, 2001, FairCom, Gaithersburg, MD, USA), the Human Metabolome Database (http://www.hmdb.ca/; accessed on 12 June 2021), Wiley 9, and the in-house library, based on their retention times, mass spectra, and mass spectral fragment patterns (*m/z*) with reference to standard compounds analyzed under identical conditions. Significantly different metabolites were identified by analysis of variance and Student’s *t*-test using Predictive Analytics SoftWare (PASW) Statistics 18 software (SPSS Inc., Chicago, IL, USA). A *p*-value < 0.05 denoted statistical significance.

## Figures and Tables

**Table 1:** Significantly different metabolites between the control group and patients with acute myeloid leukemia.

No.	Ret (min) ^a^	VIP1	VIP2	Metabolites ^b^	Unique Mass (*m/z*)	MS Fragment Pattern (*m/z*)	ID ^c^
**Amino Acids**							
1	5.48	0.26	1.54	Alanine	116	116, 73, 147, 117, 59, 100, 103, 190, 79, 148	STD/MS
2	6.65	0.30	1.17	Valine	218	73, 144, 218, 147, 100, 145, 59, 146, 219	STD/MS
3	7.19	0.49	1.08	Leucine	158	73, 158, 147, 117, 103, 205, 75, 59, 133	STD/MS
4	7.41	1.41	1.10	Isoleucine	158	73, 158, 142, 75, 147, 218, 100, 74, 159, 59	STD/MS
5	7.46	1.12	1.26	Proline	142	142, 73, 147, 75, 59, 70, 74, 66, 144, 216	STD/MS
6	7.54	0.59	1.70	Glycine	174	73, 174, 86, 147, 100, 59, 248, 133, 176	STD/MS
7	8.04	1.30	1.65	Serine	204	73, 204, 218, 100, 147, 205, 116, 188, 219	STD/MS
8	8.30	1.79	1.89	Threonine	219	73, 117, 101, 219, 218, 147, 75, 100, 129	STD/MS
9	9.06	0.55	1.29	Aminomalonic acid	218	73, 147, 218, 86, 133, 59, 174, 100, 320, 148	STD/MS
10	9.48	1.36	1.33	Methionine	176	73, 156, 147, 176, 128, 75, 100, 157, 218	STD/MS
11	9.49	1.75	1.54	Aspartic acid	232	73, 156, 232, 147, 75, 100, 157, 176, 218	STD/MS
12	9.53	1.08	1.51	5-oxoproline	156	156, 73, 147, 157, 75, 230, 258, 59, 84, 158	STD/MS
13	9.80	1.13	0.90	Cysteine	220	73, 115, 147, 100, 143, 220, 218, 116, 171	STD/MS
14	10.37	0.59	1.49	Glutamic acid	246	73, 246, 128, 84, 147, 156, 247, 100, 230, 129	STD/MS
15	10.42	2.10	1.68	Phenylalanine	192	73, 218, 192, 100, 147, 219, 193, 220, 130, 120	STD/MS
16	10.84	1.62	1.33	Asparagine	116	73, 103, 116, 147, 132, 217, 119, 117, 231, 188	STD/MS
17	12.17	1.84	1.66	Ornithine	142	73, 142, 174, 86, 59, 143, 100, 200, 175, 128	STD/MS
**Organic Acids**							
18	7.56	0.01	1.61	Succinic acid	133	147, 73, 174, 148, 79, 55, 86, 149, 247, 133	STD/MS
19	7.85	1.04	0.77	Fumaric acid	245	147, 73, 245, 148, 99, 52, 149, 133, 143, 241, 117	STD/MS
20	9.21	0.16	1.33	Malic acid	233	73, 147, 133, 55, 233, 163, 207, 148, 101	STD/MS
21	12.28	1.85	1.51	Citric acid	273	73, 147, 273, 117, 129, 133, 211, 148	STD/MS
**Fatty Acids and Lipids**							
22	7.97	0.47	1.46	Nonanoic acid	215	73, 117, 204, 215, 55, 132, 129, 131, 218, 147	MS
23	9.30	1.73	1.28	Hexanedioic acid	115	73, 100, 115, 147, 75, 117, 128, 111, 243	MS
24	10.55	1.90	1.41	Dodecanoic acid	257	73, 117, 129, 132, 257, 131, 145, 211	STD/MS
25	12.32	1.60	1.28	Myrisitic acid	117	73, 117, 147, 129, 132, 285, 133, 211	STD/MS
26	14.64	0.22	1.20	Palmitic acid	313	75, 117, 73, 55, 132, 129, 145, 57, 69, 313	STD/MS
27	17.26	2.12	1.56	Stearic acid	341	117, 73, 75, 132, 55, 129, 145, 131, 341, 133	STD/MS
**Sugars and Sugar Alcohols**							
28	9.42	0.50	1.05	Saccharide 1	217	73, 147, 217, 103, 117, 205, 133, 189, 129	MS
29	12.55	1.85	1.36	Saccharide 2	217	73, 147, 217, 191, 129, 103, 218, 133, 117, 101	MS
**Others**							
30	5.61	1.92	1.46	Hydroxylamine	146	73, 133, 146, 119, 59, 147, 86, 79, 88, 74, 155	STD/MS
31	5.74	2.16	1.60	2-Hydroxybutyric acid	133	73, 131, 147, 75, 66, 74, 148, 133, 132, 81, 149	MS
32	6.30	1.61	1.27	Monomethylphosphate	241	241, 79, 73, 163, 133, 242, 211, 135, 243	MS
33	7.29	0.76	1.12	Phosphoric acid	211	73, 299, 133, 74, 300, 75, 193, 207, 59, 314, 211	STD/MS
34	9.60	1.60	1.26	Ethylphosphate	299	73, 147, 156, 79, 84, 299, 211, 133, 155, 315, 343	MS
35	9.82	1.04	0.93	Creatinine	115	115, 73, 143, 100, 147, 116, 171, 329, 114, 144	STD/MS
**Unknowns**							
36	4.82	1.21	1.03	N.I. 1	152	73, 207, 79, 208, 123, 152, 93, 50, 295, 209	‒ ^d^
37	4.91	1.21	1.62	N.I. 2	138	79, 50, 52, 78, 73, 69, 51, 140, 147, 77, 80, 110	‒
38	6.59	0.10	1.64	N.I. 3	228	73, 144, 228, 110, 69, 77, 58, 134, 184, 74, 147	‒
39	8.93	1.31	1.28	N.I. 4	128	73, 147, 128, 75, 59, 100, 115, 350, 129, 133	‒
40	9.08	0.27	1.38	N.I. 5	232	73, 147, 232, 100, 59, 133, 148, 233, 131, 155	‒

^a^ Retention time; ^b^ metabolites selected based on variable importance in projection (VIP > 1.0) scores using the partial least squares-discriminant analysis model; ^c^ identification; ^d^ not detected. STD/MS, comparison with standard compounds analyzed under the same conditions and comparison of mass spectra with that in Human Metabolome Database, National Institutes of Standards and Technology library, and Wiley 9 database.

**Table 2:** Differentially expressed metabolites between the patients categorized based on their causative acute myeloid leukemia (AML) genetic variation.

No.	Ret (min) ^a^	VIP1	VIP2	Metabolites ^b^	Unique Mass (*m/z*)	MS Fragment Pattern (*m/z*)	ID ^c^	*p*-Value			
								Experimental Group ^d^	RUNXvsFLT ^e^	RUNX vs Others ^f^	FLT vs Others ^g^
**Amino Acids**											
1	5.48	1.22	1.09	Alanine	116	116, 73, 147, 117, 59, 100, 103, 190, 79, 148	STD/MS	0.373	0.137	0.889	0.347
2	6.65	1.70	1.24	Valine	218	73, 144, 218, 147, 100, 145, 59, 146, 219	STD/MS	0.238	0.135	0.294	0.836
3	7.19	1.16	0.90	Leucine	158	73, 158, 147, 117, 103, 205, 75, 59, 133	STD/MS	0.513	0.255	0.654	0.652
4	7.41	1.11	0.86	Isoleucine	158	73, 158, 142, 75, 147, 218, 100, 74, 159, 59	STD/MS	0.526	0.421	0.308	0.774
6	7.54	0.37	1.61	Glycine	174	73, 174, 86, 147, 100, 59, 248, 133, 176	STD/MS	0.050	0.746	0.020 *	0.070
8	8.30	0.86	1.14	Threonine	219	73, 117, 101, 219, 218, 147, 75, 100, 129	STD/MS	0.351	0.244	0.722	0.248
9	9.06	0.75	1.30	Aminomalonic acid	218	73, 147, 218, 86, 133, 59, 174, 100, 320, 148	STD/MS	0.196	0.264	0.177	0.183
17	12.17	1.90	1.45	Ornithine	142	73, 142, 174, 86, 59, 143, 100, 200, 175, 128	STD/MS	0.101	0.102	0.133	0.443
**Organic Acids**											
42	5.89	1.72	1.27	Pyruvic acid	220	73, 147, 133, 59, 100, 86, 89, 220, 148, 103, 235	STD/MS	0.221	0.121	0.361	0.564
18	7.56	0.53	1.28	Succinic acid	247	147, 73, 174, 148, 79, 55, 86, 149, 247, 133	STD/MS	0.196	0.970	0.139	0.096
19	7.85	1.65	1.43	Fumaric acid	245	147, 73, 245, 148, 99, 52, 149, 133, 143, 241, 117	STD/MS	0.144	0.087	0.644	0.260
21	12.28	0.75	1.19	Citric acid	273	73, 147, 273, 117, 129, 133, 211, 148	STD/MS	0.244	0.571	0.211	0.287
**Fatty Acids and Lipids**											
53	6.13	1.31	1.00	Hydroxyvaleric acid	145	73, 79, 145, 147, 130, 128, 148, 146, 133, 131	MS	0.412	0.328	0.361	0.634
22	7.97	1.58	1.16	Nonanoic acid	215	73, 117, 204, 215, 55, 132, 129, 131, 218, 147	MS	0.303	0.177	0.429	0.639
43	11.86	1.75	1.28	Glycerophosphate	299	73, 299, 147, 357, 101, 103, 133, 129, 211	MS	0.214	0.088	0.232	0.779
26	14.64	0.10	1.03	Palmitic acid	313	75, 117, 73, 55, 132, 129, 145, 57, 69, 313	STD/MS	0.452	0.286	0.996	0.078
**Sugars and Sugar Alcohols**											
28	9.42	1.10	1.13	Saccharide 1	217	73, 147, 217, 103, 117, 205, 133, 189, 129	MS	0.361	0.245	0.949	0.344
55	11.39	1.03	0.76	Xylitol	103	73, 103, 217, 147, 117, 129, 205, 218, 133, 243	STD/MS	0.613	0.445	0.559	0.502
29	12.55	1.95	1.46	Saccharide 2	217	73, 147, 217, 191, 129, 103, 218, 133, 117, 101	MS	0.374	0.365	0.669	0.069
**Others**											
46	4.98	1.35	1.09	1,3 Propanediol	115	147, 73, 115, 130, 66, 59, 79, 148, 177, 131, 103	MS	0.335	0.326	0.155	0.620
30	5.61	1.14	0.88	Hydroxylamine	146	73, 133, 146, 119, 59, 147, 86, 79, 88, 74, 155	STD/MS	0.512	0.407	0.336	0.764
48	6.97	1.22	0.89	Urea	189	147, 189, 73, 171, 66, 74, 148, 99, 75, 59, 87, 100	STD/MS	0.509	0.298	0.508	0.961
33	7.29	0.85	1.42	Phosphoric acid	211	73, 299, 133, 74, 300, 75, 193, 207, 59, 314, 211	STD/MS	0.117	0.724	0.124	0.034 *
34	9.60	0.59	1.09	Ethylphosphate	299	73, 147, 156, 79, 84, 299, 211, 133, 155, 315, 343	MS	0.351	0.355	0.552	0.181
35	9.82	0.95	1.14	Creatinine	115	115, 73, 143, 100, 147, 116, 171, 329, 114, 144	STD/MS	0.326	0.268	0.067	0.345
**Unknowns**											
56	4.45	0.07	1.30	N.I. 9	117	73, 117, 147, 66, 118, 59, 148, 81, 133, 149	‒ ^h^	0.188	0.573	0.150	0.110
36	4.82	1.73	1.26	N.I. 1	152	73, 207, 79, 208, 123, 152, 93, 50, 295, 209	‒	0.218	0.148	0.175	0.998
49	4.87	1.47	1.13	N.I. 6	123	123, 93, 55, 73, 125, 95, 79, 103, 75, 124	‒	0.309	0.257	0.188	0.734
37	4.91	1.50	1.15	N.I. 2	138	79, 50, 52, 78, 73, 69, 51, 140, 147, 77, 80, 110	‒	0.285	0.248	0.165	0.703
39	8.93	1.09	0.84	N.I. 4	128	73, 147, 128, 75, 59, 100, 115, 350, 129, 133	‒	0.531	0.252	0.445	0.769
40	9.08	1.56	1.60	N.I. 5	232	73, 147, 232, 100, 59, 133, 148, 233, 131, 155	‒	0.067	0.045 *	0.793	0.156
51	9.96	1.13	0.85	N.I. 8	247	73, 147, 129, 75, 247, 157, 203, 299, 349	‒	0.545	0.351	0.177	0.916

^a^ Retention time; ^b^ metabolites selected based on variable importance in projection (VIP > 1.0) scores using the partial least squares–discriminant analysis model; ^c^ identification; ^d^ significantly different metabolites between initial diagnosis patient subgroups (one-way ANOVA); ^e^ significantly different metabolites between RUNX1-RUNX1T1 and FLT3-ITD/TKD groups *(t*-test); ^f^ significantly different metabolites between RUNX1-RUNX1T1 and others groups (*t*-test); ^g^ significantly different metabolites between FLT3-ITD/TKD and others groups (*t*-test); ^h^ not detected. STD/MS, comparison with standard compounds analyzed under the same conditions and comparison of mass spectra with that in Human Metabolome Database, National Institutes of Standards and Technology library, and Wiley 9 database. * means a significantly different metabolites compared to the two patient groups.

## Data Availability

The data presented in this study are available within the article and Supplementary Materials.

## Supplementary Materials

The following are available online at https://www.mdpi.com/article/10.3390/metabo11090586/s1, Hierarchical clustering of metabolites among the acute myeloid leukemia initial diagnosis, relapse, and remission patient groups. Figure S1: Each dot on the PLS–DA plot shows the average metabolites of each leukemia patients. Figure S2: Orthogonal partial least squares-discriminant analysis (OPLS–DA) score plots derived from gas chromatography-time of flight-mass spectrometry analytical dataset for (A) control and initial diagnosis, (B) control and remission, and (C) control and relapse patient groups, Table S1: Baseline characteristics of the study participants, Table S2: Clinical course of the study participants. Table S3: Clinical information of the study participants.
