# Supplementary material for: Metabolic Profiling during Acute Myeloid Leukemia Progression Using Paired Clinical Bone Marrow Serum Samples
Source: Metabolites. 2021 Aug 31;11(9):586. doi: 10.3390/metabo11090586 (PMC8471543; doi:10.3390/metabo11090586)
Supplement: Supplementary file 1 [file metabolites-11-00586-s001.zip › Kim et al_Supplemental_figures_09-14.pdf]

Supplemental Figures

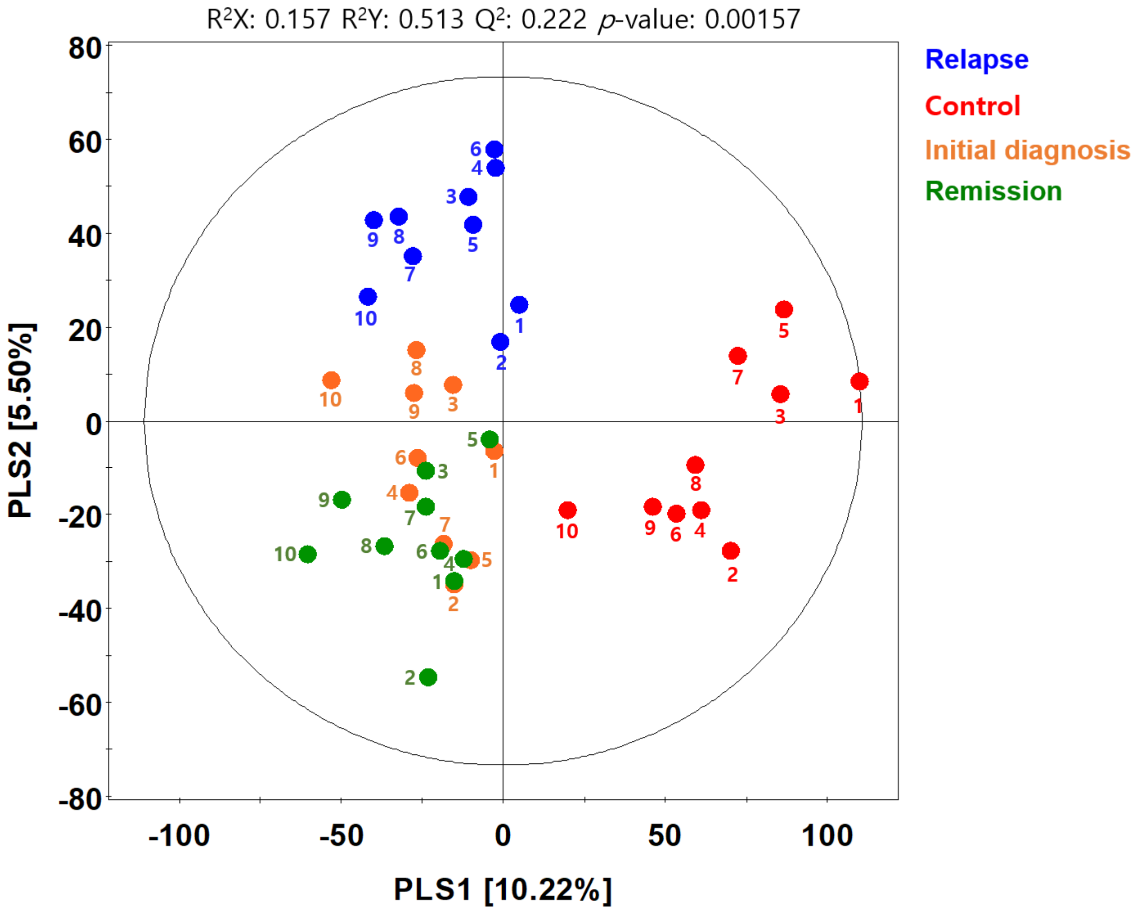

Figure S1. Each dot on the PLS-DA plot shows the average metabolites of each leukemia patient. The same number indicates that it is obtained from a sample of the same patient, and the color of the dot indicates the patient's clinical state. Red dots, control group; orange dots, initial diagnosis group; green dots, remission group; blue dots, relapse group.

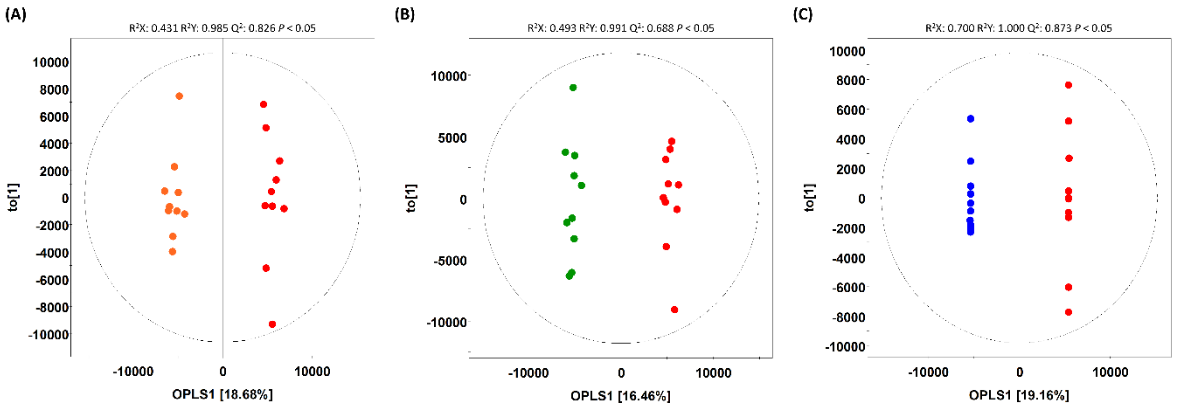

Figure S2. The OPLS-DA score plot derived from the GC-TOF-MS dataset identified distinct patterns by OPLS1. (**A**) Differences between the control and initial diagnosis groups. (**B**) Differences between the control and remission groups. (**C**) Differences between the control and relapse groups.
