# Supplementary figures and images for: Metabolic Profiling during Acute Myeloid Leukemia Progression Using Paired Clinical Bone Marrow Serum Samples
Source: Metabolites. 2021 Aug 31;11(9):586. doi: 10.3390/metabo11090586 (PMC8471543; doi:10.3390/metabo11090586)

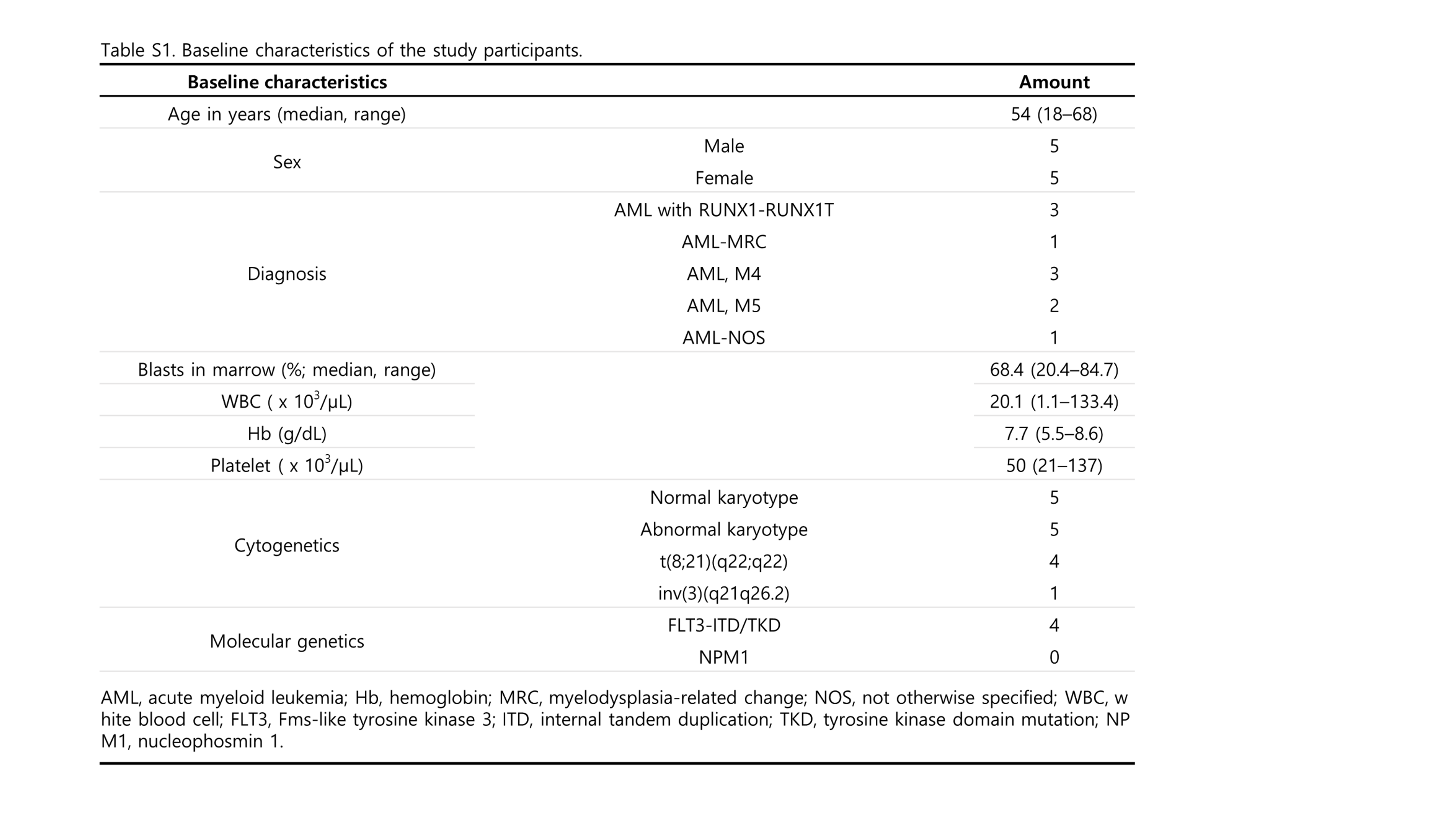

Supplement: Supplementary file 1 [file metabolites-11-00586-s001.zip › Table S1.TIF]

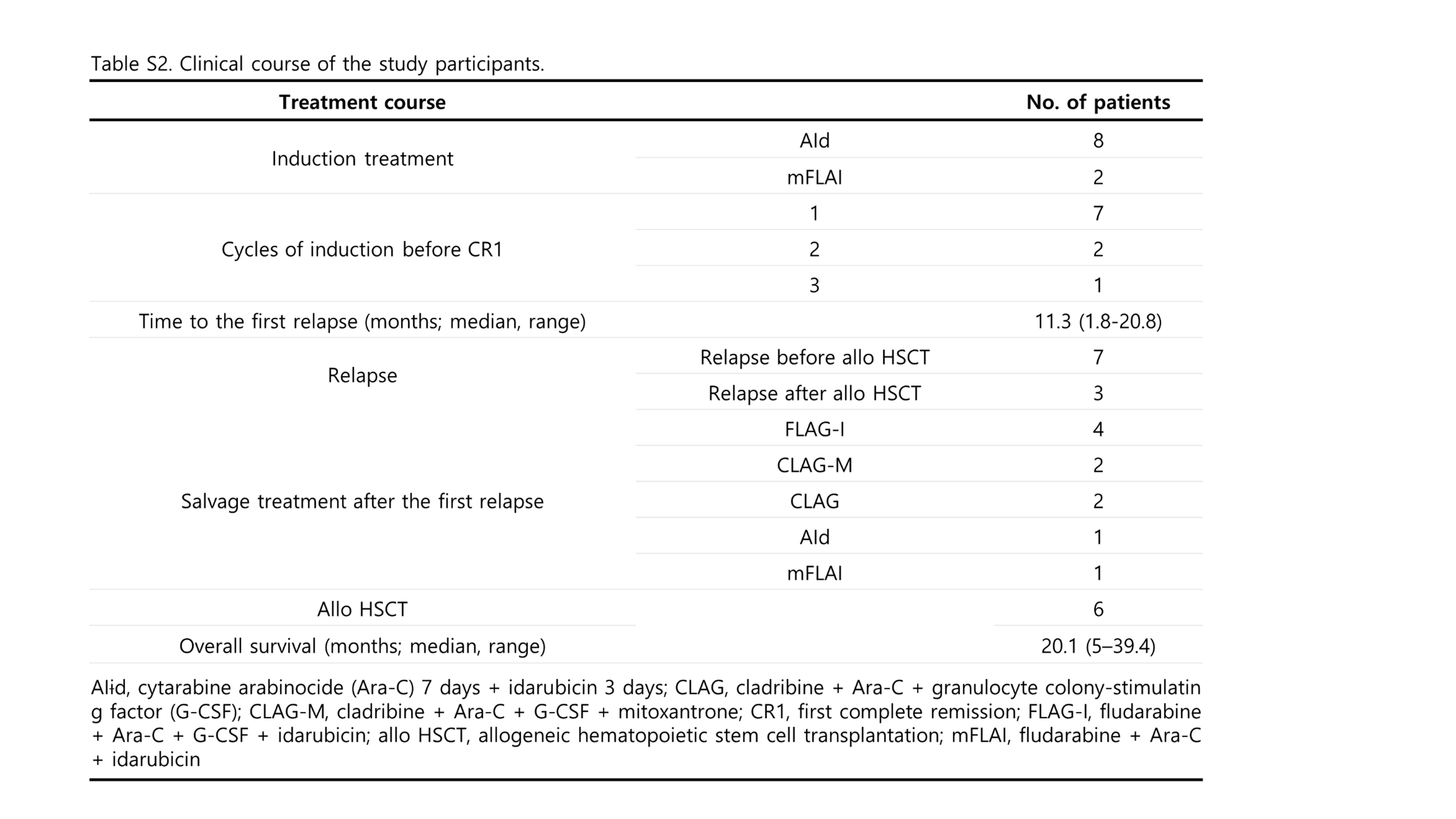

Supplement: Supplementary file 1 [file metabolites-11-00586-s001.zip › Table S2.TIF]

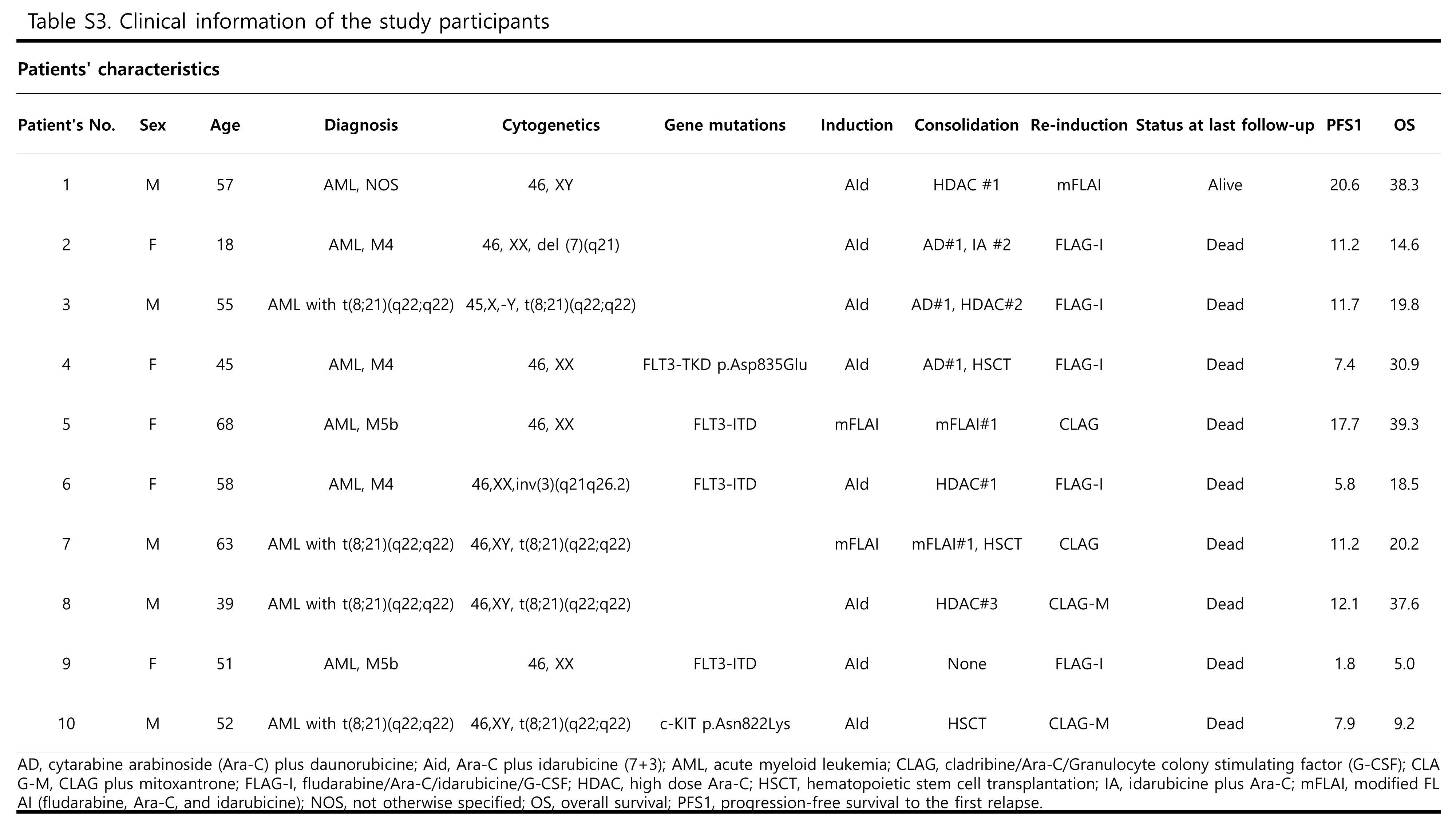

Supplement: Supplementary file 1 [file metabolites-11-00586-s001.zip › TableS3.tif]
